# Editorial: The effect of fitness on cognitive function and development in adolescents and old adults from lifespan neuroscience perspective

**DOI:** 10.3389/fnhum.2022.1033828

**Published:** 2022-09-28

**Authors:** Guo-Xin Ni, Gao-Xia Wei, Xiang-Ping Chu, Anke Ninjia Karabanov

**Affiliations:** ^1^Department of Rehabilitation Medicine, The First Affiliated Hospital of Xiamen University, Xiamen, China; ^2^Chinese Academy of Sciences Key Laboratory of Behavioral Science, Institute of Psychology, Beijing, China; ^3^Department of Biomedical Sciences, University of Missouri-Kansas City, Kansas City, MO, United States; ^4^Section for Movement and Neuroscience, Department of Nutrition, Exercise and Sports, University of Copenhagen, Copenhagen, Denmark

**Keywords:** fitness, cognitive function, cognitive development, adolescence, elderly

This Research Topic (RT) focused on the impact of fitness on cognitive function and development in adolescents and elderly adults from the standpoint of lifespan neuroscience. Adolescent brain development is characterized by multimodal integration of brain anatomical features and function, according to accumulating evidence. The elderly, on the other hand, suffer from age-related cognitive deterioration. Fitness may be a major factor influencing brain growth and cognitive performance throughout these two critical times for neurological development. It is a multidimensional notion that includes cardiovascular fitness, muscular strength and endurance, flexibility, and body composition, as well as motor fitness (balance, agility, speed, power, and coordination). Adolescents and older people may be responsive to intervention trials aimed at improving fitness levels, such as outdoor activities, cardiovascular exercise, and mind-body practice since brain network integration and neural efficiency are rapidly altered during this period. This RT investigates how fitness influences structural and functional brain development, particularly cognitive functions, and emotional health in adolescents and elderly people, as well as training strategies that may assist cognitive progress throughout this time. It includes a clinical trial (1) and original research papers (4) on the influence of fitness on cognitive function and development in adolescents and elderly adults from a lifespan neuroscience viewpoint.

Due to the long maturation of the prefrontal cortex, childhood is regarded as a highly sensitive and critical period for executive function development (Michel et al., 2018; Chen et al., 2020). All subcomponents of executive function grow significantly between the ages of 7 and 12, commensurate with increased gray matter density in the brain (Bidzan-Bluma and Lipowska, 2018). During this period, executive function development is more responsive to ambient and external stimuli, such as physical activities (Ludyga et al., 2016; Takacs and Kassai, 2019). Age-related cognitive decline in the elderly includes impaired sustained attention, memory loss, or impaired executive function, as well as dramatic changes in similar brain tissues (e.g., prefrontal cortex, posterior parietal cortex, superior temporal cortex) to support or compensate for high-order cognition function. Physical activity prevents age-related cognitive deficits by improving executive functioning and memory (Hötting and Röder, 2013). This RT aims to expand our understanding of the impact of physical activities in influencing anatomical and functional brain development in adolescents and older individuals.

## The relationship between physical activities and cognition function in adolescents and young adults

Xu et al. (a) investigated the relationship between the frequency of basketball training and executive functions in a sample of 60 boys aged 6–8. They were separated into three groups based on their training frequency: the low-frequency group (once a week), the high-frequency group (at least twice a week), and the control group (no training experience). The Stop-signal task, the N-back task, and the switching task were used to measure executive functioning (inhibitory control, working memory, and cognitive flexibility). The authors suggested that regular basketball training, especially with higher frequency is beneficial to working memory and cognitive flexibility.

In terms of training experience, Xu et al. (b) recruited 60 children aged 8–12 and divided them into two groups: short-term (< 12 months) and long-term (more than 12 months). Results showed that children trained for over 1 year have better performance in cognitive flexibility and working memory than those trained in < 1 year. The findings suggested that training experience is positively associated with executive functions.

Song et al. investigated an association between obesity and cardiorespiratory fitness concerning their potential effects on cognitive flexibility in young adults. Four groups of 140 young individuals were formed: normal weight with high cardiorespiratory fitness, obese with high cardiorespiratory fitness, normal weight with low cardiorespiratory fitness, and obese with low cardiorespiratory fitness. The task-switching test was performed. Their findings confirmed that cardiorespiratory fitness has beneficial effects on cognitive flexibility, attentional resource allocation, and sensory evaluation in young adults.

## The effect of physical activities on molecular and structural brain change in late-middle-aged adults and patients with chronic obstructive pulmonary disease

To investigate the cognitive effect and underlying mechanisms of aerobic exercise (AE), computerized cognitive training (CCT), and a combined (COMB) groups in comparison to a waitlist control group, Castells-Sánchez et al. conducted a 12-week (5 days per week-45 min per day) multi-domain, single-blind, proof-of-concept randomized controlled trial. Brain volume was measured by structural MRI and plasma biomarkers (BDNF, TNF, HGF, ICAM-1, SDF1-) were measured. Changes in ICAM-1 and SDF1-α were negatively associated with changes in physical activity outcomes in AE and COMB groups. Brain volume changes were found in the CCT showing a significant increase in precuneus volume. This study demonstrated significant early molecular and brain volume alterations linked to lifestyle modifications.

Shen et al. explored the impact of integrating routine treatment with an 8-week Tai Chi Chuan (TCC) intervention on the clinical symptoms of 20 patients with Chronic Obstructive Pulmonary Disease (COPD). Clinical symptoms were evaluated by Chronic Obstructive Pulmonary Symptom Assessment Scale (CAT) and Modified Dyspnea Scale (mMRC) at baseline and after treatment. Resting-state MRI scan was also performed with multiline T2-weighted echo-planar imaging (EPI) to acquire their functional images before and after the treatment. The patient's clinical symptoms significantly improved after the 8-week incorporation of normal treatment with TCC practice. In the right inferior frontal gyrus (IFG), right middle frontal gyrus, bilateral cingulate cortex, bilateral precuneus, and right precentral gyrus, imaging analysis revealed lower Degree of Centrality (DC) in COPD patients. Additionally, further analysis revealed a positive correlation between the increased CAT and the decreased DC in the right IFG. These findings provided neurological support for treating COPD rehabilitation practice with mind-body practice based on Chinese culture to some extent.

In conclusion, the studies included in this RT show that fitness is a beneficial strategy to alter cognitive function and development in children and elderly people across the lifespan. Our hope is that this RT will inspire researchers to design and implement fitness training strategies aiming at assisting cognitive progress in both older persons and adolescents.

## Author contributions

All authors listed have made a substantial, direct, and intellectual contribution to the work and approved it for publication.

## Funding

G-XN was supported by the National Key Research and Development Program (2020YFC2006703).

## Conflict of interest

The authors declare that the research was conducted in the absence of any commercial or financial relationships that could be construed as a potential conflict of interest.

## Publisher's note

All claims expressed in this article are solely those of the authors and do not necessarily represent those of their affiliated organizations, or those of the publisher, the editors and the reviewers. Any product that may be evaluated in this article, or claim that may be made by its manufacturer, is not guaranteed or endorsed by the publisher.

## References

[B1] Bidzan-Bluma I. Lipowska M. (2018). Physical activity and cognitive functioning of children: a systematic review. Int. J. Environ. Res. Public Health. 15, 800. 10.3390/ijerph15040800 29671803 PMC5923842

[B2] Chen P. Wang D. Shen H. Yu L. Gao Q. Mao L.. (2020). Physical activity and health in Chinese children and adolescents: expert consensus statement (2020). Br. J. Sports Med. 54, 1321–1331. 10.1136/bjsports-2020-102261 32471813 PMC7606574

[B3] Hötting K. Röder B. (2013). Beneficial effects of physical exercise on neuroplasticity and cognition. Neurosci. Biobehav. Rev. 37(9 Pt B), 2243–2257. 10.1016/j.neubiorev.2013.04.005 23623982

[B4] Ludyga S. Gerber M. Brand S. Holsboer-Trachsler E. Puhse U. (2016). Acute effects of moderate aerobic exercise on specific aspects of executive function in different age and fitness groups: a meta-analysis. Psychophysiology 53, 1611–1626. 10.1111/psyp.12736 27556572

[B5] Michel E. Molitor S. Schneider W. (2018). Differential changes in the development of motor coordination and executive functions in children with motor coordination impairments. Child Neuropsychol. 24, 20–45. 10.1080/09297049.2016.1223282 27623087

[B6] Takacs Z. K. Kassai R. (2019). The efficacy of different interventions to foster children's executive function skills: a series of meta-analyses. Psychol. Bull. 145, 653–697. 10.1037/bul0000195 31033315

